# Out of sync: an unexpected rhythm in the NICU

**DOI:** 10.1093/ehjcr/ytaf282

**Published:** 2025-06-04

**Authors:** Murad Almasri, Abdelrahman F Masri, Nick Von Bergen

**Affiliations:** Pediatric Cardiology Department, Arkansas Children’s Hospital, 1 Children’s Way, Little Rock, AR 72202, USA; Pediatric Cardiology Department, Arkansas Children’s Hospital, 1 Children’s Way, Little Rock, AR 72202, USA; Pediatric Cardiology Department, The University of Wisconsin School of Medicine and Public Health, 1675 Highland Avenue, Madison, WI 53792, USA

## Clinical vignette

**Figure 1:**
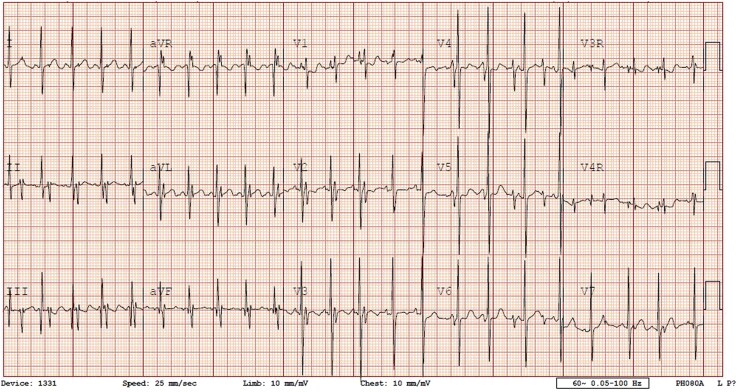
The ECG mentioned in the vignette.

A cardiologist was sitting in his office reviewing the day’s stack of ECGs, when a 15-lead tracing caught his attention (*Figure 1*). It was obtained from a 14-week-old infant who was referred from an outside hospital and is currently in the NICU. The indication was brief and unremarkable: ‘Pre-operative screening’.

**ECG:**

**1. What is the diagnosis based on the history and ECG findings?**
  - Atrial bigeminy with intermittent fusion beats
  - Lead misplacement
  - Two separate rhythms from thoracopagus (shared thorax) conjoined twins
  - Junctional rhythm with variable atrial capture
  - Electromagnetic interference from NICU monitoring equipment

The correct answer is **C**

The ECG shows two clearly distinct QRS morphologies alternating in a regular pattern. One is taller and narrower; the other is broader and with lower amplitude, suggesting two independent cardiac rhythms with two separate hearts in a thoracopagus conjoined twin.^1^ Atrial bigeminy shows alternating normal and ectopic beats, not two regular rhythms. Lead misplacement may alter wave morphology but does not produce two distinct rates. Junctional rhythm features absent or retrograde P waves, not dual organized atrial activity. Electromagnetic interference causes erratic, noisy baselines, not structured rhythms. This ECG should raise suspicion for anatomic duplication, particularly in pre-operative NICU infants with minimal clinical data.

**2. Which of the following ECG findings indicates poor chances of a successful surgery for the diagnosis above?**
  - Single QRS complex
  - Electrical alternans
  - Sinus arrhythmia with rate variability
  - Intermittent PVCs
  - Presence of two independent sinus rhythms

The correct answer is **A**

A single QRS complex on the ECG suggests electrical fusion or shared myocardium/conduction system, making separation risky or impossible without damaging one or both hearts.^2,3^ This implies the twins’ hearts are not electrically independent. Electrical alternans usually indicates pericardial effusion, not shared cardiac tissue. Sinus arrhythmia is common and benign, especially in infants. PVCs can occur in healthy or sick hearts; they don’t reflect shared cardiac conduction. Though surgical outcomes for separation of thoracopagus conjoined twins are poor,^4^ two independent rhythms indicate separate conduction systems, which could represent a favourable sign for separation, at least with respect to cardiac anatomy.

**3. Which of the following is most indicative of an imminent risk of cardiac compromise in the surgery done for the diagnosis above?**
  - Increased QTc
  - Flattened T waves
  - Sudden loss of one QRS complex
  - Bradycardia below 100 bpm
  - Left axis deviation

The correct answer is **C**

In conjoined twins with two separate hearts, the presence of two QRS complexes on the pre-operative ECG signifies electrical and functional independence. During surgical separation, if one QRS complex suddenly disappears, it suggests that one heart has stopped conducting or contracting. This could be due to accidental ligation of coronary vessels, ventricular fibrillation not visible on the combined thorax, conduction tissue disruption, or myocardial ischaemia during dissection. Other options (A, B, D, E) may indicate metabolic or conduction issues but do not directly reflect sudden electrical silence of an entire heart.

## Data Availability

No new data were generated or analysed in support of this research.

## Notes

**Consent:** Patient consent was obtained for this submission.

**Funding:** None declared.
